# Genome-wide meta-analysis of short-tandem repeats for Parkinson’s disease risk using genotype imputation

**DOI:** 10.1093/braincomms/fcae146

**Published:** 2024-04-23

**Authors:** Olena Ohlei, Kimberly Paul, Susan Searles Nielsen, David Gmelin, Valerija Dobricic, Vivian Altmann, Marcel Schilling, Jeff M Bronstein, Andre Franke, Michael Wittig, Laura Parkkinen, Johnni Hansen, Harvey Checkoway, Beate Ritz, Lars Bertram, Christina M Lill

**Affiliations:** Lübeck Interdisciplinary Platform for Genome Analytics (LIGA), University of Lübeck, 23562 Lübeck, Germany; Department of Neurology, UCLA David Geffen School of Medicine, Los Angeles, CA 90095, USA; Department of Neurology, Washington University School of Medicine, St. Louis, MO 63108, USA; Lübeck Interdisciplinary Platform for Genome Analytics (LIGA), University of Lübeck, 23562 Lübeck, Germany; Lübeck Interdisciplinary Platform for Genome Analytics (LIGA), University of Lübeck, 23562 Lübeck, Germany; Lübeck Interdisciplinary Platform for Genome Analytics (LIGA), University of Lübeck, 23562 Lübeck, Germany; Forensic Genetics Division, Instituto-Geral de Perícias do Rio Grande do Sul, Porto Alegre, RS 90230-010, Brazil; Lübeck Interdisciplinary Platform for Genome Analytics (LIGA), University of Lübeck, 23562 Lübeck, Germany; Gene Regulation of Cell Identity, Regenerative Medicine Program, Bellvitge Institute for Biomedical Research (IDIBELL), L’Hospitalet del Llobregat, Barcelona 0890x, Spain; Department of Epidemiology, University of California Los Angeles (UCLA), Fielding School of Public Health, Los Angeles, CA 90095, USA; Department of Neurology, UCLA David Geffen School of Medicine, Los Angeles, CA 90095, USA; Brain Research Institute, University of California Los Angeles (UCLA), Los Angeles, CA 90095, USA; Institute of Clinical Molecular Biology, Christian-Albrechts-University of Kiel, 24105 Kiel, Germany; Institute of Clinical Molecular Biology, Christian-Albrechts-University of Kiel, 24105 Kiel, Germany; Nuffield Department of Clinical Neurosciences, Oxford Parkinson’s Disease Centre, University of Oxford, Oxford OX1 3PT, UK; Danish Cancer Institute, Danish Cancer Society, 2100 Copenhagen, Denmark; Herbert Wertheim School of Public Health, University of California San Diego, La Jolla, CA 92093, USA; Department of Neurology, UCLA David Geffen School of Medicine, Los Angeles, CA 90095, USA; Brain Research Institute, University of California Los Angeles (UCLA), Los Angeles, CA 90095, USA; Lübeck Interdisciplinary Platform for Genome Analytics (LIGA), University of Lübeck, 23562 Lübeck, Germany; Institute of Epidemiology and Social Medicine, University of Münster, 48149 Münster, Germany; School of Public Health, Imperial College, Ageing Epidemiology Research Unit, London SW71, UK

**Keywords:** genome-wide association study, Parkinson’s disease, microsatellites, short-tandem repeats, DNA methylation

## Abstract

Idiopathic Parkinson’s disease is determined by a combination of genetic and environmental factors. Recently, the first genome-wide association study on short-tandem repeats in Parkinson’s disease reported on eight suggestive short-tandem repeat-based risk loci (*α* = 5.3 × 10^−6^), of which four were novel, i.e. they had not been implicated in Parkinson’s disease risk by genome-wide association analyses of single-nucleotide polymorphisms before. Here, we tested these eight candidate short-tandem repeats in a large, independent Parkinson’s disease case–control dataset (*n* = 4757). Furthermore, we combined the results from both studies by meta-analysis resulting in the largest Parkinson’s disease genome-wide association study of short-tandem repeats to date (*n* = 43 844). Lastly, we investigated whether leading short-tandem repeat risk variants exert functional effects on gene expression regulation based on methylation quantitative trait locus data in human ‘post-mortem’ brain (*n* = 142). None of the eight previously reported short-tandem repeats were significantly associated with Parkinson’s disease in our independent dataset after multiple testing correction (*α* = 6.25 × 10^−3^). However, we observed modest support for short-tandem repeats near *CCAR2* and *NCOR1* in the updated meta-analyses of all available data. While the genome-wide meta-analysis did not reveal additional study-wide significant (*α* = 6.3 × 10^−7^) short-tandem repeat signals, we identified seven novel suggestive Parkinson’s disease short-tandem repeat risk loci (*α* = 5.3 × 10^−6^). Of these, especially a short-tandem repeat near *MEIOSIN* showed consistent evidence for association across datasets. *CCAR2*, *NCOR1* and one novel suggestive locus identified here (*LINC01012*) emerged from colocalization analyses showing evidence for a shared causal short-tandem repeat variant affecting both Parkinson’s disease risk and *cis* DNA methylation in brain. Larger studies, ideally using short-tandem repeats called from whole-sequencing data, are needed to more fully investigate their role in Parkinson’s disease.

## Teaser

Ohlei *et al*.’s updated genome-wide association study on short-tandem repeats (STRs) in 43 844 Parkinson’s disease cases and controls supported two (*CCAR2* and *NCOR1*) of eight previously reported risk STRs. Seven novel suggestive risk loci were identified, including a STR near *MEIOSIN*. Several STRs may act via DNA methylation changes.

## Graphical Abstract

**Graphical Abstract:**
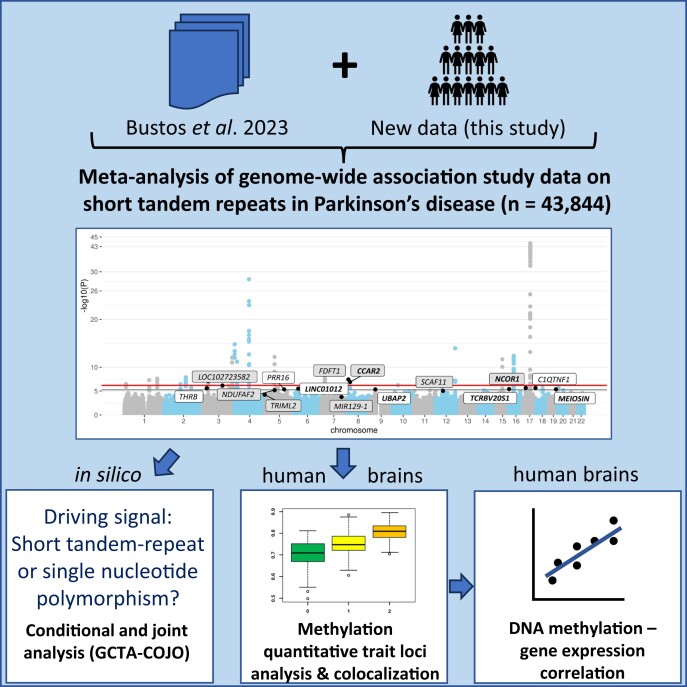

## Introduction

Idiopathic Parkinson’s disease is the second most common neurodegenerative disease after Alzheimer’s disease and is determined by a combination of genetic and environmental risk factors.^1^ The to date largest genome-wide association study (GWAS) on single-nucleotide polymorphisms (SNPs) by Nalls *et al.*^2^ reported 90 SNPs that were independently associated with Parkinson’s disease risk. However, common SNPs account only for 16–36% of the total genetic heritability of the disease,^2^ suggesting that other genetic variants play a role in Parkinson’s disease susceptibility. One example of previously understudied genetic variants is short-tandem repeats (STRs, also known as microsatellites), i.e. repeating sequence motifs in the human genome of 1–6 nucleotides in length.^3^ Recently, Bustos *et al*.^4^ performed a GWAS using STRs in 16 642 patients with Parkinson’s disease and 22 445 controls from the International Parkinson’s Disease Genetics Consortium (IPDGC) dataset and identified 34 index STRs that showed study-wide significant association with Parkinson’s disease risk (defined in that study^4^ as *α* = 5.34 × 10^−6^), eight of which exerted risk effects independent of SNPs in the respective regions. Four of these eight STRs represented putative novel Parkinson’s disease risk loci, namely *NCOR1, NDUFAF2*, *TRIML2* and *MIR129-1,*^4^ while the other four independent STRs were located in risk loci already known by SNP-based GWAS.^2^ These exciting results await replication in independent datasets. Thus, we tested these eight candidate Parkinson’s disease risk STRs in a large, independent case–control dataset (2419 cases and 2338 controls). Furthermore, we combined the results from both studies by meta-analysis, resulting in the largest (*n* = 43 844) Parkinson’s disease STR GWAS to date. Lastly, we investigated whether leading Parkinson’s disease risk STRs may exert functional effects on gene expression regulation via DNA methylation (DNAm).

## Materials and methods

### Datasets and data processing

Detailed methods are given in the Supplementary material. Briefly, we generated genome-wide SNP data using the Global Screening Array (v1 with shared custom content) in three population-based case–control datasets from the USA [the Parkinson Environment Gene (PEG) study^5^ and a study among Group Health Cooperative (GHC) members^6^] and Denmark (the Parkinson’s disease in Denmark [PASIDA] study),^7^ resulting in an effective sample size of 2419 patients with Parkinson’s disease and 2338 controls after quality control (QC; Supplementary Table 1). Using the reference haplotype panel from Saini *et al*.,^8^ we imputed 445 180 unique STR positions based on 457 368 genotyped SNPs and split unique STR positions with multi-allelic variants into single bi-allelic variants. After QC, this resulted in 1 097 832 unique STR identifiers available for the subsequent statistical analyses. For the meta-analysis of our data with those of Bustos *et al*.,^4^ the total sample size was 19 061 Parkinson’s disease cases and 24 783 controls.

### Statistical analyses

Genetic association analyses were performed in PLINK (https://www.cog-genomics.org/plink/2.0/). For the replication arm of the study, statistical significance was defined using Bonferroni’s correction for eight tests (*α* = 0.05/8 = 6.25 × 10^−3^). We performed genome-wide association analyses [i.e. logistic regression analyses adjusting for sex and the first four principal components (PCs)] with Parkinson’s disease status in each dataset separately followed by fixed-effect meta-analysis of 1 044 914 STRs overlapping in at least two datasets (Supplementary Fig. 1**)**. To define an appropriate study-wide significance threshold,^9^ we estimated the number of PCs explaining 95% of the variance in our STR data (*n* = 78 968 PCs) and used this number as denominator for Bonferroni’s correction (i.e. *α*= 0.05/78 968 = 6.3 × 10^−7^). Since this threshold is nearly one order of magnitude more stringent than the study-wide significance threshold applied by Bustos *et al*.^4^ (*α* = 5.3 × 10^−6^), we defined the latter as ‘study-wide suggestive’ evidence for association for our study. All reported *P*-values are two-sided. For novel STRs identified in our data, to assess the independency of the STR signal on SNPs, we performed GCTA-COJO (conditional and joint association) analyses (https://yanglab.westlake.edu.cn/software/gcta/#Overview) using STR- and SNP-based GWAS summary statistics and performed logistic regression analyses on the index STR while conditioning on the top SNP in the same region. Furthermore, to assess potential functional effects of the eight reported^4^ as well as the additional potential Parkinson’s disease risk STR loci we identified here, we investigated their colocalization with *cis* methylation quantitative trait locus (meQTL) effects in 142 entorhinal cortex brain samples from the longitudinal, prospective ‘Oxford Project to Investigate Memory and Aging’ study.^10,11^ To this end, we imputed the STR genotypes from genome-wide SNP data in these brain samples using the same pipeline as described above. After QC of both STR genotypes and DNAm data, we performed *cis* meQTL association analyses for each STR (±1 Mb). Subsequently, we performed colocalization analyses for the eight reported^4^ and seven newly identified suggestive risk STRs and the most significant CpG within the respective locus from the *cis* meQTL association analyses using the R-package coloc (v5.2.2).^12^ The resulting *P*-values were false discovery rate (FDR)-controlled (FDR = 0.05). Lastly, we probed for correlation between DNAm and gene expression for those STR GWAS signals that colocalized using RNA sequencing (RNA-seq) data generated in the same samples using methods described previously.^7^ Briefly, the normalized RNA-seq data of the nearest gene located upstream of the respective CpG were correlated with the corresponding DNAm signal, using the Spearman method in R’s cor.test function. The resulting *P*-values were FDR-controlled (FDR = 0.05).

## Results

**Figure 1:**
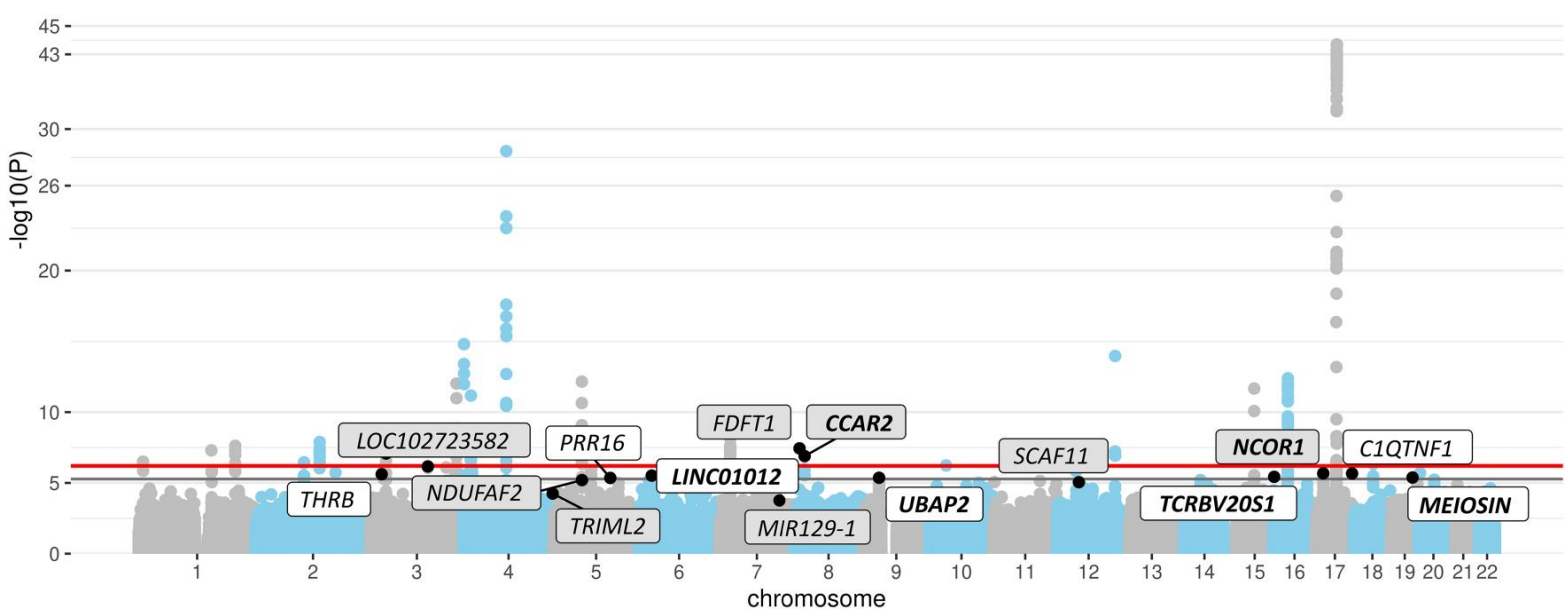
**Manhattan’s plot of genome-wide meta-analysis results of STRs in Parkinson’s disease risk.** This Manhattan plot depicts the results of the genome-wide association analysis of 1 044 914 STRs in 19 061 Parkinson’s disease cases and 24 783 controls based on logistic regression analyses followed by fixed-effect meta-analysis of all included datasets. The *y*-axis displays the **−**log10 of the *P*-value (‘**−**log10(*P*)’). The upper horizontal bold line represents the study-wide significance threshold of *α* = 6.3 × 10^−7^, and the lower horizontal bold line represents the study-wide suggestive significance threshold of *α* = 5.3 × 10^−6^. STR signals that are independent from SNPs and show at least study-wide suggestive evidence for association with Parkinson’s disease status in the previous and/or in the current study are annotated with the name of the nearest gene. Gene names for previously reported study-wide suggestive or significant STR signals are displayed in grey boxes, and gene names for novel STR signals are displayed in white boxes. Gene names for which meta-analysis results increase in significance when combining our new data with the data from the IPDGC are displayed in bold.

Of the eight top STR loci that showed association independent from Parkinson’s disease risk SNPs in the study by Bustos *et al*.,^4^ none showed statistically significant evidence for association (*α* = 6.25 × 10^−3^) in our independent datasets after accounting for multiple testing. However, five of these eight STRs showed the same direction of effect estimates in our independent datasets comprising 2419 Parkinson’s disease cases and 2338 controls (Table 1, Supplementary Table 2**)**. In particular, *CCAR2* nearly reached nominal evidence of association with Parkinson’s disease (*P* = 0.0676), and we observed a similar magnitude of association for *CCAR2* and *NCOR1* as in the previous study.^4^ Upon meta-analysis of our data with those reported in the original study,^4^ only the STRs in these two genes increased in significance, as effect estimates for the other three were clearly weaker than in the original study.^4^ Nonetheless, overall, two of the eight loci (*CCAR2* and *FDFT1*) still showed study-wide evidence (*α* = 6.3 × 10^−7^; using the empirical threshold determined in the current study) and three more (*NCOR1*, *LOC102723582* and *NDUFAF2*) showed study-wide suggestive evidence (*α* = 5.3 × 10^−6^; using the threshold suggested by Bustos *et al*.^4^). This includes two (*NCOR1* and *NDUFAF2*) of the four novel STR-based Parkinson’s disease risk loci reported by Bustos *et al*.^4^ The other two novel loci (*MIR129-1* and *TRIML2*) were no longer significant (*α* = 5.3 × 10^−6^) upon meta-analysis with effect estimates pointing into the opposite direction in the original versus our datasets (Fig. 1, Table 1, Supplementary Table 2).

**Table 1:** Genetic association results for the eight previously reported and seven novel putative Parkinson’s disease STR risk loci

STR position	Nearest gene	Effect allele	MAF_new_	OR_IPDGC_	*P*_IPDGC_	OR_new_	*P*_new_	OR_meta_	*P*_meta_	Directions	Indep. signal	*P*_meQTL_	H4
*(a) Replication evidence for previously reported primary STR loci*													
3:122 146 661	*LOC102723582*	[A]12C[A]6	0.13	1.12	6.52E−07	1.05	3.85E−01	1.11	**6.87E**−**07**	−−−−	STR	7.71E−04	0.4500
4:189 000 404	*TRIML2*	TT[A]12	0.05	1.31	1.44E−07	0.87	1.67E−01	1.20	5.66E−05	+++−	STR	9.73E−03	0.0101
5:60 437 492	*NDUFAF2*	AA[TGAA]6	0.43	0.77	6.94E−08	0.95	1.85E−01	0.87	**6.32E−06**	++−+	STR	7.63E−07	0.0340
7:127 793 488	*MIR129-1*	[T]14G	0.25	0.86	2.79E−07	1.05	2.78E−01	0.91	1.73E−04	+−−+	STR	3.83E−13	**0**.**9045**
8:11 696 990	*FDFT1*	TCTACT[A]13	0.25	0.89	1.96E−09	1.01	7.68E−01	0.91	**3.62E−08**	−−++	STR	1.23E−07	**0**.**9866**
8:22 464 976	***CCAR2***	TAGGG[T]20GATG	0.34	0.91	6.91E−07	0.92	6.76E−02	0.91	**1.28E−07**	−+++	STR	1.23E−31	**0**.**9790**
12:46 452 915	*SCAF11*	AAGCAAGCA	0.42	0.92	5.19E−06	0.97	5.29E−01	0.93	8.91E−06	−+−+	STR	3.35E−04	0.1409
17:15 941 750	***NCOR1***	[T]10	0.46	0.93	3.77E−06	0.95	2.35E−01	0.93	**2.10E−06**	++++	STR	7.42E−11	**0**.**8356**
*(b) Novel suggestive primary STR associations (P < 5.3E*−*6)*													
3:24 310 875	*THRB*	[CA]2[CG]6[CA]18[T]3[A]2G	0.04	*-*	*-*	0.60	**2.43E−06**	0.60	**2.43E−06**	+++?	STR	2.94E**−**04	0.1642
5:120 435 533	*PRR16*	CTC[A]19T	0.08	*-*	*-*	0.70	**4.45E−06**	0.70	**4.45E−06**	−−−?	STR	2.02E−02	0.0580
6:27 666 933	***LINC01012***	[A]9G[A]8	0.09	0.87	**7.26E−06**	0.90	1.68E−01	0.87	**3.05E−06**	++++	*?*	6.62E−09	**0**.**8284**
9:34 054 301	***UBAP2***	[T]13	0.39	1.07	**8.96E−06**	1.06	2.04E−01	1.10	**4.15E−06**	−−−−	*?*	4.07E−03	0.3404
16:1 977 617	***TCRBV20S1***	[A]12	0.29	0.91	**1.26E−05**	0.93	1.11E−01	0.91	**3.71E−06**	++−+	*?*	9.59E−09	0.0711
17:77 019 776	*C1QTNF1*	[GC]6G[CA]18	0.04	*-*	*-*	1.68	**2.16E−06**	1.68	**2.16E−06**	+++?	STR	7.50E−07	0.2577
19:46 243 112	***MEIOSIN***	[T]11	0.69	1.09	**6.67E−05**	1.11	**1.94E−02**	1.10	**4.15E−06**	−−−−	STR	1.77E−19	0.1722

This table displays all signals from the IPDGC that are due to independent STR signals (upper part of the table) and all study-wide suggestive primary STR signals from the updated meta-analyses combining the IPDGC data with our newly generated STR data. Bold gene names highlight the STR-based signals for which the updated meta-analysis results improved in significance. Bolded meta-analysis *P*-values indicate at least study-wide suggestive evidence for association (*α* = 5.3E−6).

STR position, position for the first nucleotide of the STR (hg19); MAF, minor allele frequency; OR, odds ratio; new, the newly generated STR data independent of the IPDGC data (datasets: PEG, PASIDA, GHC); *P*, *P*-value; meta, results of fixed-effect meta-analysis; direction, directions of effect in the individual datasets included in the meta-analysis provided in the following study order: PEG, PASIDA, GHC, IPDGC (see Material and Methods for details on these datasets); Indep. signal, result of the ‘conditional and joint association’ (COJO) and/or conditional analyses assessing whether STR signal is independent from single-nucleotide polymorphisms (‘STR’ denotes independence and ‘?’ analyses could not be performed); meQTL, methylation quantitative trait locus.

Next, we investigated whether any novel Parkinson’s disease risk STR signals emerged upon combining the previously published^4^ with our newly generated genome-wide STR data. However, we did not observe any study-wide significant (*α* = 6.3 × 10^−7^) primary STR signals that were independent from SNP associations in the same region. While our analyses revealed one STR (*EFNA*/*GBA1*, location of the first base pair: 1:155 089 886) that achieved study-wide significance [odds ratio (OR) = 2.15, *P* = 5.02 × 10^−8^] and had not been analysed in the previous study^4^ (Supplementary Table 3), this novel signal became non-significant upon conditioning on the well-established^2^ Parkinson’s disease risk SNP rs35749011 (*r*^2^ = 0.96) near *GBA1* in our data (*P*_cond_ = 0.637). Likewise, the corresponding SNP- and STR-based analyses in COJO were non-significant (*P*_COJO_ = 6.4 × 10^−1^, Supplementary Table 4), suggesting that the STR association signal was due to the association signal of the Parkinson’s disease risk SNP. Furthermore, we observed seven novel study-wide suggestive (*α* = 5.3 × 10^−6^, i.e. using the significance threshold applied in the original study^4^) STR signals (*THRB*, *PRR16*, *LINC01012*, *UBAP2*, *TCRBV20S1*, *C1QTNF1* and *MEIOSIN**)***, three of which (in or near the genes *THRB*, *PRR16* and *C1QTNF1*) had not been analysed in the original study^4^ (Table 1, Supplementary Table 3). Of the remaining four STRs, especially the association of the STR near *MEIOSIN* showed consistent evidence for association with *P* < 0.05 in both the previous^4^ and the dataset newly generated for this study (**Table 1**). Conditional and/or COJO analyses could be performed on the first four novel regions (the remaining three could not be included owing to the unavailability of SNP-based data from Bustos *et al*.^4^). Interestingly, these analyses indicated that the STRs (but not SNPs) represented the primary association signals in all four regions (Supplementary Table 4).

When we performed colocalization analysis of the eight previously reported^4^ STRs with genetic association data from STR-based *cis* meQTL analyses using DNAm profiles derived from human entorhinal cortex, we found a high posterior probability (PP.H4 > 0.8) for the presence of a shared causal variant for both Parkinson’s disease risk and *cis* meQTL effects for four of the eight previously reported loci (*CCAR2*, *NCOR1*, *MIR129-1* and *FDFT1*). Of the novel suggestive STR loci we identified, one (*LINC01012*) appeared to share a causal variant for both Parkinson’s disease risk and *cis* meQTL effects (**Table 1**, Supplemental Table 5). These results suggest that the above STRs might unfold their potential Parkinson’s disease risk effects via affecting DNAm levels. To assess whether the differential methylation observed at these loci may elicit expression changes of neighbouring genes, we performed exploratory correlation analyses between DNAm and gene expression data available in the same brain samples. While these analyses did not reveal any significant correlation after FDR control, we observed one nominally significant correlation for methylation at CpG cg09414187 and *CENPV* expression in the *NCOR1* locus (*P* = 0.028, Supplemental Table 5).

## Discussion

In this study, we performed the first independent assessment of the main findings from an STR-based GWAS recently published for Parkinson’s disease .^4^ By combining these earlier results with our novel data on 4757 Parkinson’s disease cases and controls, we substantially increased the power of the previous Parkinson’s disease STR GWAS. Furthermore, we investigated alterations in DNAm in ‘post-mortem’ brains as one potential functional mechanism underlying STR association signals.

While none of the eight previously reported^4^ STRs showed statistically significant association with Parkinson’s disease risk in our independent data following multiple testing correction, we observed modest support for two of the previously reported STR loci (*CCAR2* and *NCOR1*) in the updated meta-analyses. Furthermore, our genome-wide GWAS meta-analysis across all available data did not reveal any study-wide significant (*α* = 6.3 × 10^−7^) STR signals. However, we identified seven novel suggestive STR Parkinson’s disease risk loci (*THRB*, *PRR16*, *LINC01012*, *UBAP2*, *TCRBV20S1*, *C1QTNF1* and *MEIOSIN*) that may be independent from nearby SNPs. Of these, an STR near *MEIOSIN* showed particularly consistent evidence for association with *P* < 0.05 in both the previous^4^ and the dataset newly generated for this study. Interestingly, both *CCAR2* and *NCOR1* as well as one of the novel putative loci identified here (*LINC01012*) also showed evidence for a shared causal STR variant affecting both Parkinson’s disease risk and *cis* DNAm in human brain, offering a possible functional basis for the observed statistical associations. It should be noted that correlating DNAm levels with gene expression data available in the same dataset for these colocalizing CpG signals yielded only one nominally significant result, specifically for the gene *CENPV* in the *NCOR1* locus. However, these results were not significant after FDR adjustment. A lack of observed expression QTLs for meQTL-specific GWAS colocalizations has been described in previous studies (e.g. ref.^13^), which is in agreement with our negative correlation results. In addition, in the previous study,^4^ the STR in the *NCOR1 locus* was also described to show distinct *cis* expression QTL effects on several nearby genes across different brain regions, including higher expression of *TRPV2*, *NCOR1* and *ADORA2B* and lower expression of a long non-coding RNA gene (CTC-529I10.1) located near *NCOR1*. The exact—seemingly intricate—regulatory molecular mechanisms underlying the association signal of the *NCOR1* STR as well as the other STRs on Parkinson’s disease risk need to be investigated in future functional studies.

Despite numerous strengths, our study also has several potential limitations. First, while our independent dataset was well powered to validate all previously described eight STR signals with at least nominal significance, some of the non-validations of the candidate STRs assessed in this study may represent false-negative findings. Furthermore, while the combined STR GWAS dataset analysed in this study represents the largest Parkinson’s disease GWAS on STRs to date, our sample size may still be underpowered to detect subtle risk effects. Second, one commonly applied threshold to determine genome-wide significance in the context of GWAS is *α* = 5 × 10^−8^. However, this threshold captures the correlation structure for common SNPs in European genomes to keep the study-wide error rate at 5%. Other variant types (such as STRs or copy number variants) require different thresholds to achieve a study-wide error rate of 5%. To this end, we have empirically assessed the correlation structure for the STRs analysed in our study and defined the *α* level accordingly (at *α* = 6.3 × 10–7). We note that this threshold is already approximately one order of magnitude more stringent than the threshold applied by Bustos *et al*. However, even our more conservative threshold does not exclude the possibility of false positive findings inherent in our data. Third, the commonly applied STR association analysis model applied in this study and the previous study^4^ splits multi-allelic STRs into multiple bi-allelic variants. While this is a valid approximation to allow an efficient computational analysis of the underlying genome-wide data, this likely does not mirror the exact biological action of genuine risk STRs. Lastly, the interpretation of these analyses is complicated by the fact that the STR data used here and previously^4^ rely on imputations based on SNP genotypes possibly generating collinearity in the molecular data. Thus, the distinction between primary STR versus SNP effects should be reassessed using genome-wide STR profiles determined by other methods, e.g. STRs called directly from whole-genome sequencing.^14^ Future work, ideally based on genome-wide STR data not relying on SNP-based imputations, needs to validate and further characterize these findings and possibly uncover additional STR signals relevant for Parkinson’s disease susceptibility.

## Supplementary Material

fcae146_Supplementary_Data

## Data Availability

Summary statistics of the STR GWAS meta-analysis across the PEG, PASIDA and GHC datasets are publicly available at the GWAS Catalog (https://www.ebi.ac.uk/gwas/home, accession number: GCST90296681) as well as at the Zenodo platform (https://zenodo.org; study data accessible at https://doi.org/10.5281/zenodo.10069354). Subject-level data can be obtained by qualified investigators upon request to the authors (Prof. Nielsen, Prof. Ritz and Prof. Lill).
