# Supplementary material for: Genome-wide meta-analysis of short-tandem repeats for Parkinson’s disease risk using genotype imputation
Source: Brain Commun. 2024 Apr 23;6(3):fcae146. doi: 10.1093/braincomms/fcae146 (PMC11166220; doi:10.1093/braincomms/fcae146)
Supplement: fcae146_Supplementary_Data [file fcae146_supplementary_data.zip › Supplementary_Materials.docx]

**Supplementary Materials**

*Genome-wide SNP data genotyping, processing and genome-wide STR imputation:*

SNP genotyping of DNA samples from blood (PEG, GHC) or saliva (PASIDA) was performed using the Global Screening Array (GSA) v2 with shared custom content (Illumina, Inc., USA) on an iScan instrument according to the manufacturer's recommendations. Genotyping was performed at the Institute of Clinical Molecular Biology, Kiel University, Germany. After completion of all genotyping experiments, raw intensity data were processed and quality-controlled using an automated bioinformatics workflow as previously described^1,2^. Briefly, SNP genotypes from raw data were all called simultaneously using the GenomeStudio software v. 2.0.2 (Illumina). Quality control (QC) included removing of ambiguous SNPs, flipping and swapping alleles to align to human genome assembly GRCh37/hg19 before imputation. We excluded es well SNPs with minor allele frequency (MAF) ≥ 0.01, SNPs deviating from Hardy-Weinberg equilibrium (HWE) in controls (α=5×10^-6^). Furthermore, samples were excluded in case of low genotyping efficiency (<98%), discrepancies between genetic and recorded sex, duplicated DNA sampling, cryptic relatedness (Pi-hat ≥0.05), and excess heterozygosity (mean±6SD). Finally, STR genotype imputation based on SNP genotypes was performed with Beagle software^3^ using the 1000 Genomes SNP-STR Haplotype Panel (including 445,725 STR markers from 1,916 individuals)^4^. STRs were split from multi-allelic variants to single biallelic variants using bcftools tool (v. 1.9)^5^. We excluded STRs with low imputation quality score (DR2 <0.3), minor allele frequency (MAF) <1%, and statistically significant deviations from HWE in control individuals (alpha=5×10^-6^). To correct for population stratification, principal component analysis (PCA) was performed in PLINK (v2.0)^6^ using SNP genotype data with the five “superpopulation” codes from the 1000 Genomes project^7^ as reference. Only samples clustering to the “CEU” population cluster were retained. Subsequently, outliers were determined by re-performing the PCA and removed based on a false discovery rate (FDR)=0.05. Both PCAs were performed on a subset of 88,510 LD-pruned (threshold: r^2^<0.2; command: --indep-pair 1500 150 0.2), SNPs using PLINK (--pca).

*Statistical analysis:*

To compute the STR-level association with PD risk, we used logistic regression models in PLINK (v2.0)^6^ (command: --glm), including sex and the first 4 principal components of the PCA as covariates. GWAS analyses were performed for each dataset separately. Meta-analyses of our results and those of Bustos et al., 2023^8^ were performed using the inverse variance weighting model as implemented in PLINK (v1.9)^6^. To account for multiple testing, we estimated the number of independent STR loci based on the number of PCs that explained 95% of the variance in the data. This number was used to adjust the genome-wide significance threshold according to the Bonferroni method (i.e., α=0.05/78968=6.33×10^-7^). We performed conditional analyses using the **GCTA tool (**v1.94)^9^. For STR showing at least nominal significance (p<0.05) in our datasets we performed conditional analysis (--cojo-cond) based on the top 90 STRs from the 2019 GWAS of Nalls et al.^10^ as well as based on the top associated SNPs in an +/- 1MB region from our dataset. Furthermore, we performed conditional analysis (--cojo-slct, --cojo-p 5.3×10^-6^) using both SNP and STR summary level data from our datasets.

To determine the functional processes of the identified risk loci, we used the R package coloc (v.5.2.2)^11^ and investigated common causal effects that influence both PD risk and methylation in entorhinal cortex (EC) tissue. Methylation data were derived from participants of the longitudinal, prospective Oxford Project to Investigate Memory and Aging (OPTIMA) study and have been described in detail elsewhere^12,13^. We imputed the STRs genotypes to EC samples based on SNP genotypes. After QC of both STR genotypes and DNAm patterns, we performed *cis* methylation quantitate trait locus (meQTL) association analyses (SNP-CpG distance within 1 Mb) using the R package MatrixEQTL^14^. For each top STR in a PD risk locus, we selected the top associated CpG based on lowest p value and performed a colocalization analysis of the PD case-control and *cis* mQTL results within a 1 Mb region using R-package coloc (v.5.2.2)^11^ .


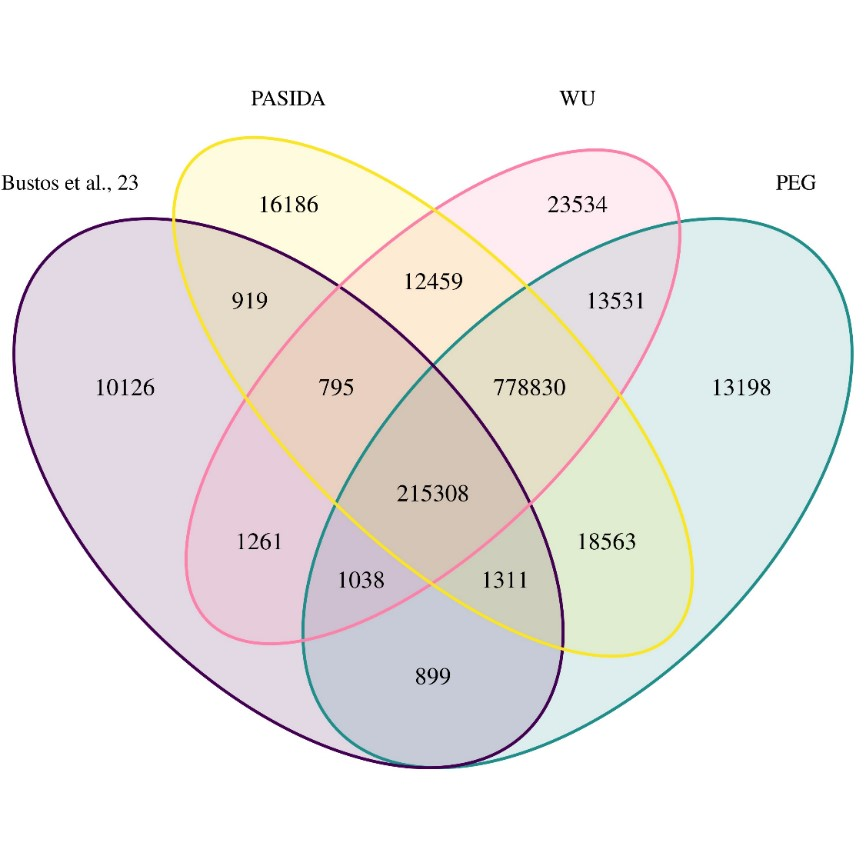


**Supplemental Figure 1.** Venn diagram of quality-controlled STR variants overlapping across the datasets PASIDA (n=3205), PEG (n=1172), GHC (n=380), and the dataset of Bustos et al.^8^

**Supplementary References**

[1] Hong S, Dobricic V, Ohlei O, et al. TMEM106B and CPOX are genetic determinants of cerebrospinal fluid Alzheimer’s disease biomarker levels. *Alzheimer’s Dement*. 2021 Oct;17(10):1628-1640.

[2] Hong S, Prokopenko D, Dobricic V, et al. Genome-wide association study of Alzheimer’s disease CSF biomarkers in the EMIF-AD Multimodal Biomarker Discovery dataset. *Transl Psychiatry*. 2020;10(1):403.

[3] Browning BL, Zhou Y, Browning SR. A One-Penny Imputed Genome from Next-Generation Reference Panels. *Am J Hum Genet*. 2018;103(3):338-348.

[4] Saini S, Mitra I, Mousavi N, Fotsing SF, Gymrek M. A reference haplotype panel for genome-wide imputation of short tandem repeats. *Nat Commun*. 2018;9(1):1-11.

[5] Li H. A statistical framework for SNP calling, mutation discovery, association mapping and population genetical parameter estimation from sequencing data. *Bioinformatics*. 2011;27(21):2987.

[6] Purcell S, Neale B, Todd-Brown K, et al. PLINK: a tool set for whole-genome association and population-based linkage analyses. *Am J Hum Genet*. 2007;81(3):559-575.

[7] Auton A, Abecasis GR, Altshuler DM, et al. A global reference for human genetic variation. *Nature*. 2015;526(7571):68-74.

[8] Bustos BI, Billingsley K, Blauwendraat C, et al. Genome-wide contribution of common short-tandem repeats to Parkinson’s disease genetic risk. *Brain*. 2023;146(1):65-74.

[9] Yang J, Ferreira T, Morris AP, et al. Conditional and joint multiple-SNP analysis of GWAS summary statistics identifies additional variants influencing complex traits. *Nat Genet 2012 444*. 2012;44(4):369-375.

[10] Nalls MA, Blauwendraat C, Vallerga CL, et al. Identification of novel risk loci, causal insights, and heritable risk for Parkinson’s disease: a meta-analysis of genome-wide association studies. *Lancet Neurol*. 2019;18(12):1091-1102.

[11] Wallace C. Eliciting priors and relaxing the single causal variant assumption in colocalisation analyses. *PLOS Genet*. 2020;16(4):e1008720.

[12] Dobricic V, Schilling M, Schulz J, et al. ARTICLE Differential microRNA expression analyses across two brain regions in Alzheimer’s disease. *Transl Psychiatry*. 2022 Aug 29;12(1):352

[13] Sommerer Y, Dobricic V, Schilling M, Ohlei O, et al. Entorhinal cortex epigenome-wide association study highlights four novel loci showing differential methylation in Alzheimer’s disease. *Alzheimers Res Ther*. 2023 May 6;15(1):92

[14] Shabalin AA. Matrix eQTL: ultra fast eQTL analysis via large matrix operations. *Bioinformatics*. 2012;28(10):1353-1358.
